# *Pseudomonas aeruginosa* mannose-sensitive hemagglutinin inhibits proliferation and invasion via the PTEN/AKT pathway in HeLa cells

**DOI:** 10.18632/oncotarget.9467

**Published:** 2016-05-19

**Authors:** Tie-qiu Yin, Xuan Ou-yang, Fang-yan Jiao, Lu-ping Huang, Xu-dong Tang, Bi-qiong Ren

**Affiliations:** ^1^ The Second People's Hospital of Hunan Province, Clinical Medical College of Hunan University of Chinese Medicine, Changsha 410007, China

**Keywords:** cervical cancer, PA-MSHA, PTEN, p-AKT, p-GSK3β

## Abstract

We investigated the effects of *Pseudomonas aeruginosa* mannose-sensitive hemagglutinin (PA-MSHA) on the proliferation and invasion of human cervical cancer cell lines, as well as the molecular pathways underlying these effects. MTT cell proliferation assays revealed a time- and concentration-dependent cytotoxic effect of PA-MSHA on HeLa cells but not H8 cells. Flow cytometry with propidium iodide and annexin-V-fluorescein isothiocyanate labeling (FITC) indicated that various concentrations of PA-MSHA could induce apoptosis and G2-M cell cycle arrest in HeLa cells. PA-MSHA also impaired the migration and invasion abilities of HeLa cells in Wound healing and Transwell invasion assays. Western blot results demonstrated that PA-MSHA reduced the expression of p-AKT, p-GSK3β, BCL-2, Vimentin and β-catenin, but increased the levels of PTEN, BAD, BAX and E-cadherin in HeLa cells. Importantly, *PTEN* siRNA induced the activity of p-AKT, while PA-MSHA partly inhibited this induction, indicating that PA-MSHA may reduce the cell proliferation and invasion potential by activating PTEN and thus inhibiting the AKT pathway *in vitro*. These data suggest the potential application of PA-MSHA to the treatment of human cervical cancer.

## INTRODUCTION

Cervical cancer is a major malignancy of the female genital tract that can spread to adjacent organs and to the pelvic and para-aortic lymph nodes. Every year, over 530,000 cases are diagnosed, and over 275,000 women die from the disease worldwide [1]. Although the rate of cervical cancer has declined since 1960, this disease is still a major burden in the Asia-Pacific region [2]. Human Papilloma Virus (HPV) DNA has been detected in 90–100% of premalignant and malignant lesions of the cervical mucosa, and HPV is well known to be the etiological agent in these neoplasms [3]. However, HPV infection alone is not sufficient to transform epithelial host cells to cancer cells. Thus, other factors, such as the upregulation of oncogenes and the aberrant activation of related signaling pathways, could be involved in cervical carcinogenesis. Three methods are commonly used to treat cervical cancer: surgery, chemotherapy and radiation therapy. However, recent research has demonstrated that engineered bacteria can be used to treat cancer [4], and the development of a new treatment method for cervical cancer is feasible.

*Pseudomonas aeruginosa* is an extracellular, Gram-negative bacterium. The proliferative response of normal adult peripheral blood lymphocytes increases upon exposure to a heat-killed version of *P. aeruginosa* [5]. The *P. aeruginosa* vaccine is widely used for anti-infection and anti-inflammation purposes and for immune suppressive in anti-tumor therapies [6, 7]. Recently, a vaccine using *P. aeruginosa*-mannose-sensitive hemagglutinin (PA-MSHA) was shown to enhance the antigen-presenting function of dendritic cells by activating their proliferation and differentiation [8]. The PA-MSHA vaccine also reduced the Th2/Th1 cell ratio in a mouse model of IgA nephropathy. In addition, PA-MSHA had anti-carcinogenic activity against human hepatocarcinoma and gastric nasopharyngeal carcinoma [9, 10]. PA-MSHA may have anti-proliferative effects on breast cancer cells by inducing a death receptor-related apoptotic signaling pathway and the cell cycle arrest [11]. These findings suggest that PA-MSHA might be beneficial in cervical cancer treatment and could be a possible tool in adjuvant therapy modalities; however, the *in vitro* effects of PA-MSHA on cervical cancer cells remain unclear.

In this study, we hypothesized that PA-MSHA would inhibit the proliferation and invasion of cervical cancer cells. We characterized the anti-tumor activities of PA-MSHA *in vitro* and assessed the potential for a PA-MHSA-based vaccine for future clinical applications against cervical cancer.

## RESULTS

### PA-MSHA inhibits the growth of human cervical carcinoma (HeLa) cells

**Figure 1:**
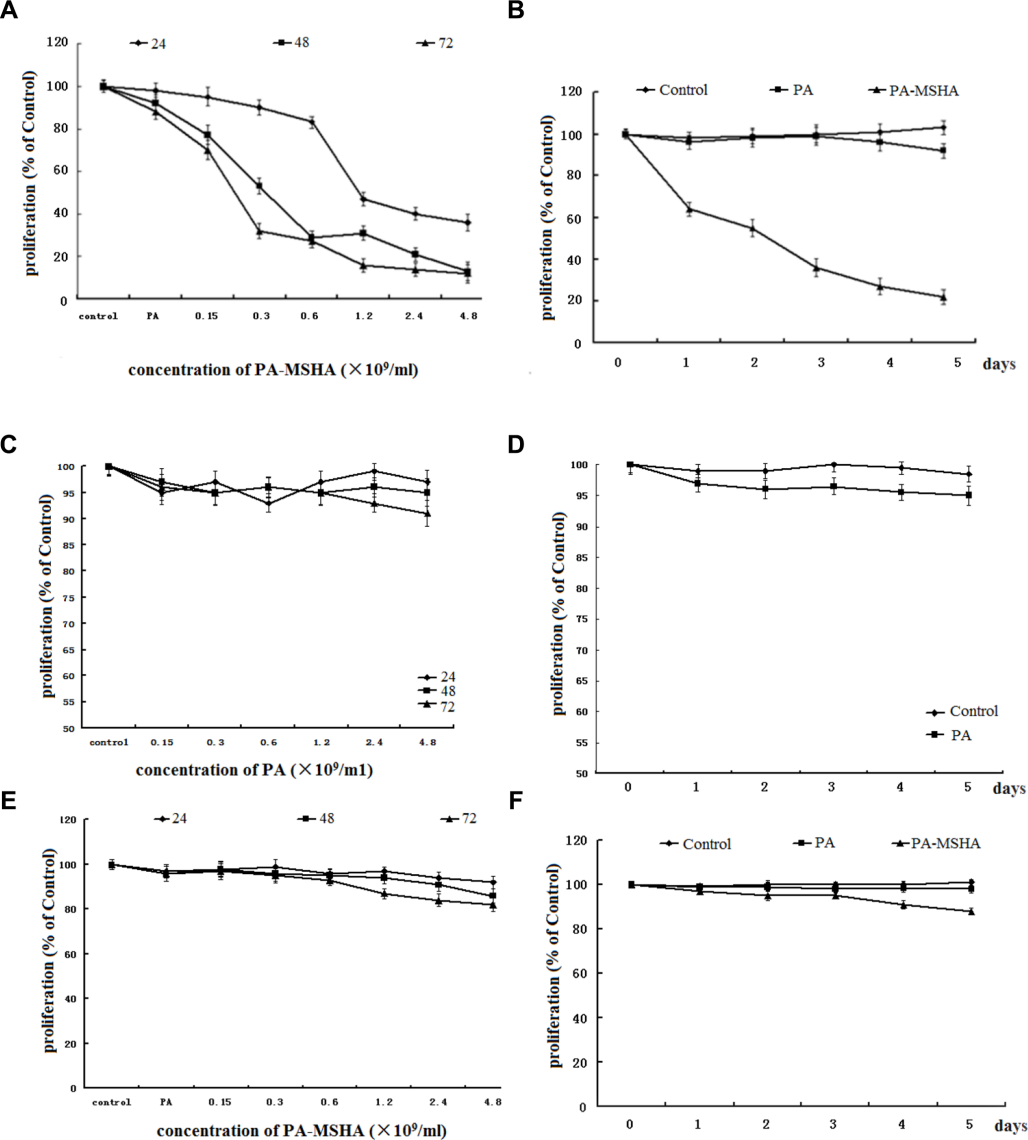
Effect of PA-MSHA or PA on cell proliferation Values are given as percentages of the values in untreated control cells. The data are presented as the averages of triplicate results from a representative experiment; bars, standard deviation (SD). (**A**) The dose-dependent effect of PA-MSHA on HeLa cell proliferation. (**B**) The time-dependent effect of PA-MSHA on HeLa cell proliferation. (**C**) The dose-dependent effect of PA on HeLa cell proliferation. (**D**) The time-dependent effect of PA on HeLa cell proliferation. (**E**) The dose-dependent effect of PA-MSHA on H8 cell proliferation. (**F**) The time-dependent effect of PA-MSHA on H8 cell proliferation.

The MTT assay revealed that exposure of HeLa cells to PA-MSHA for up to 72 h had a cumulative concentration- and time-dependent inhibitory effect on cell proliferation (Figure 1A and 1B). The negative control for PA-MSHA, exogenously added PA (Figure 1C and 1D), caused very little cell growth inhibition in HeLa cells at the same incubation concentrations and during the same time periods. However, exogenously added PA-MSHA did not inhibit the proliferation of the immortalized human cervical epithelial (H8) cell line (Figure 1E and 1F).

### PA-MSHA changes the distribution of cells across the phases of the cell cycle and induces apoptosis

**Figure 2:**
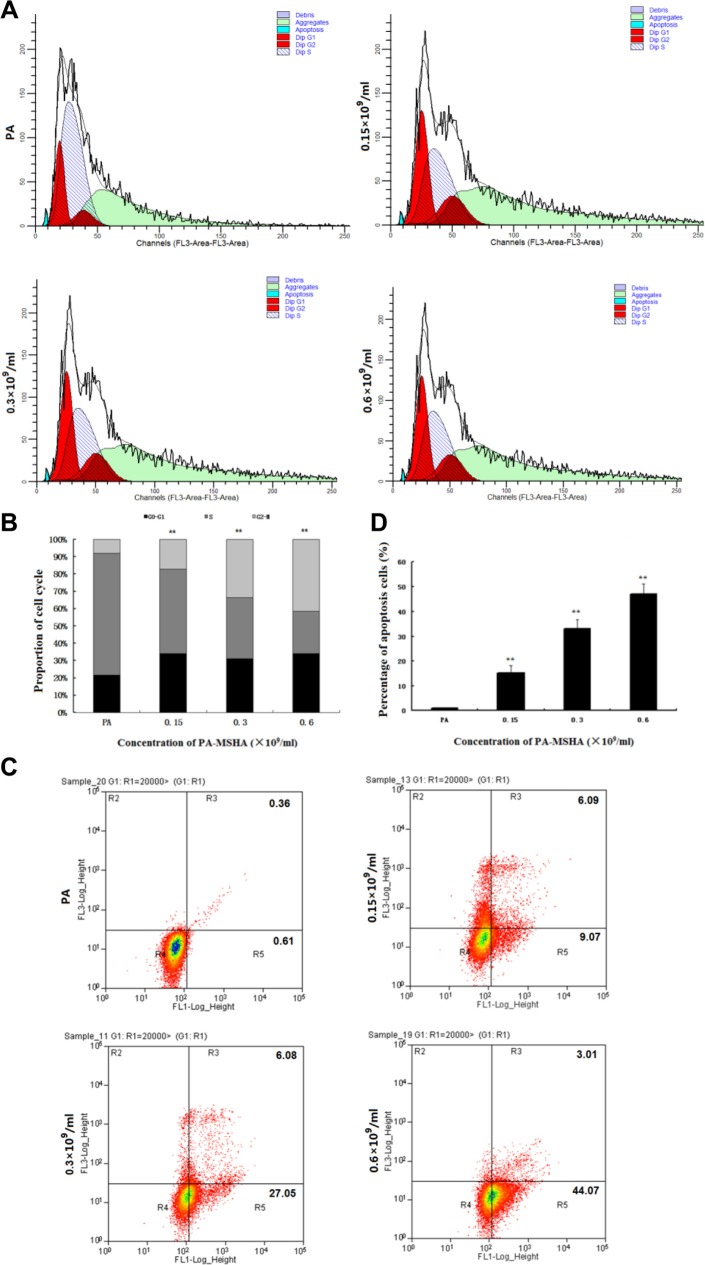
Effects of PA-MSHA on cell cycle arrest and apoptosis The distribution of HeLa cells in the three phases of the cell cycle following treatment with PA or PA-MSHA is depicted in representative plots (**A**) and as percentages (**B**). **p < 0.01 for cells treated with PA-MSHA versus PA in the G0–G1, S and G2-M phases. (**C**) The percentages of apoptotic cells were measured in HeLa cells treated with PA or PA-MSHA, which are shown in representative plots. (**D**) The percentages of apoptotic cells were measured in HeLa cells treated with PA or PA-MSHA. The data are presented as the mean ± SD (*N* = 3, **p < 0.01).

Because PA-MSHA inhibited the proliferation of HeLa cells, we investigated the mechanism by which PA-MSHA suppressed growth. Based on the results of the MTT assay, we chose three different concentrations of PA-MSHA for further investigation. Cells treated with either PA or PA-MSHA for 48 h were stained with PI and analyzed by flow cytometry. Challenging HeLa cells with increasing concentrations of PA-MSHA (0.15, 0.3 or 0.6 × 10^9^/mL) dose-dependently arrested the cell cycle at the G2/M phase, thereby reducing the proportion of cells in the S phase (Figure 2A and 2B).

To determine whether the growth inhibitory effect of PA-MSHA was associated with cell apoptosis, we performed Annexin V-PI double-staining and flow cytometry of HeLa cells. The total apoptotic rates were 15.16 ± 3.05%, 33.13 ± 3.70% and 47.08 ± 4.09% at PA-MSHA concentrations of 0.15, 0.3 and 0.6 × 10^9^/mL, respectively (Figure 2C and 2D). These results demonstrate that the inhibition of cell growth by PA-MSHA was caused by the induction of apoptosis.

### PA-MSHA inhibits the migration and invasion of HeLa cells

**Figure 3:**
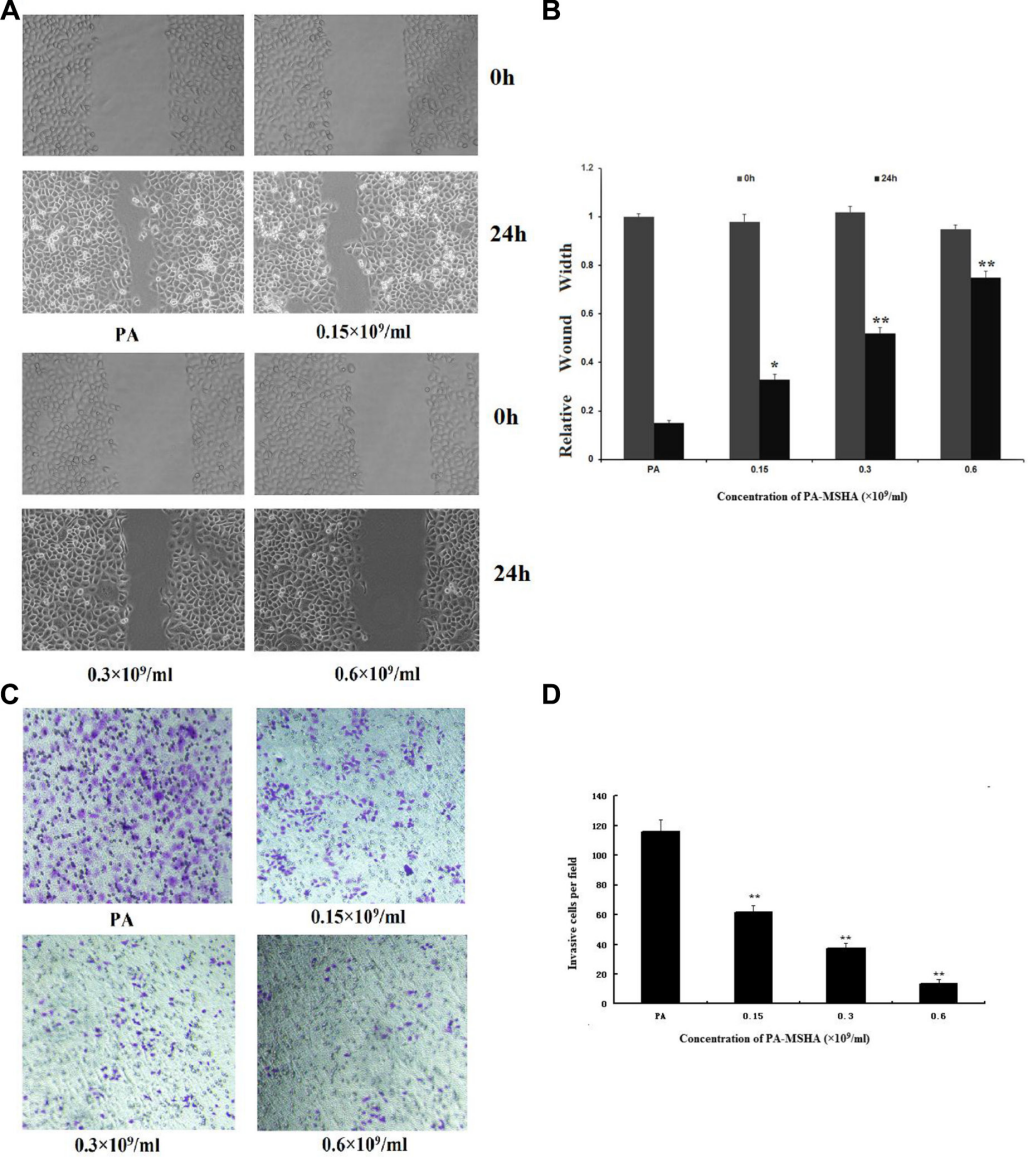
Effects of PA-MSHA on cell migration and invasion (**A**) Wound healing assay. The changes in the wound edges are illustrated with broken lines. Upper panel: PA or PA-MSHA group at 0-h timepoint. Bottom panel: PA or PA-MSHA group at 24-h timepoint. (**B**) The wound edges were measured in HeLa cells treated with PA or PA-MSHA. Data are presented as the mean ± SD (*N* = 3, **p < 0.01). (**C**) The numbers of invasive cells per field were counted in HeLa cells treated with PA or PA-MSHA, and are shown in representative pictures. (**D**) The numbers of invasive cells per field were counted in HeLa cells treated with PA or PA-MSHA. Data are presented as the mean ± SD (*N* = 3, **p < 0.01).

To investigate the effect of PA-MSHA on the cell migration ability, we performed the *in vitro* scratch wound healing assay. As shown in Figure 3A and 3B, HeLa cell migration was significantly slower in the PA-MSHA group than in the PA group. To investigate whether PA-MSHA would affect the invasive abilities of HeLa cells *in vitro*, we performed the Transwell assay with PA-MSHA- and PA-treated cells. As shown in Figure 3C and 3D, PA-MSHA-treated HeLa cells had significantly lower invasive abilities than PA-treated cells. The percentages of migratory cells were 115.57 ± 8.31%, 61.32 ± 5.11% and 37.14 ± 3.27% at PA-MSHA concentrations of 0.15, 0.3 and 0.6 × 10^9^/mL, respectively. These results suggest that PA-MSHA is an important inhibitor of migration and invasion in HeLa cells.

### Effects of PA-MSHA on apoptosis- and EMT-related proteins

**Figure 4:**
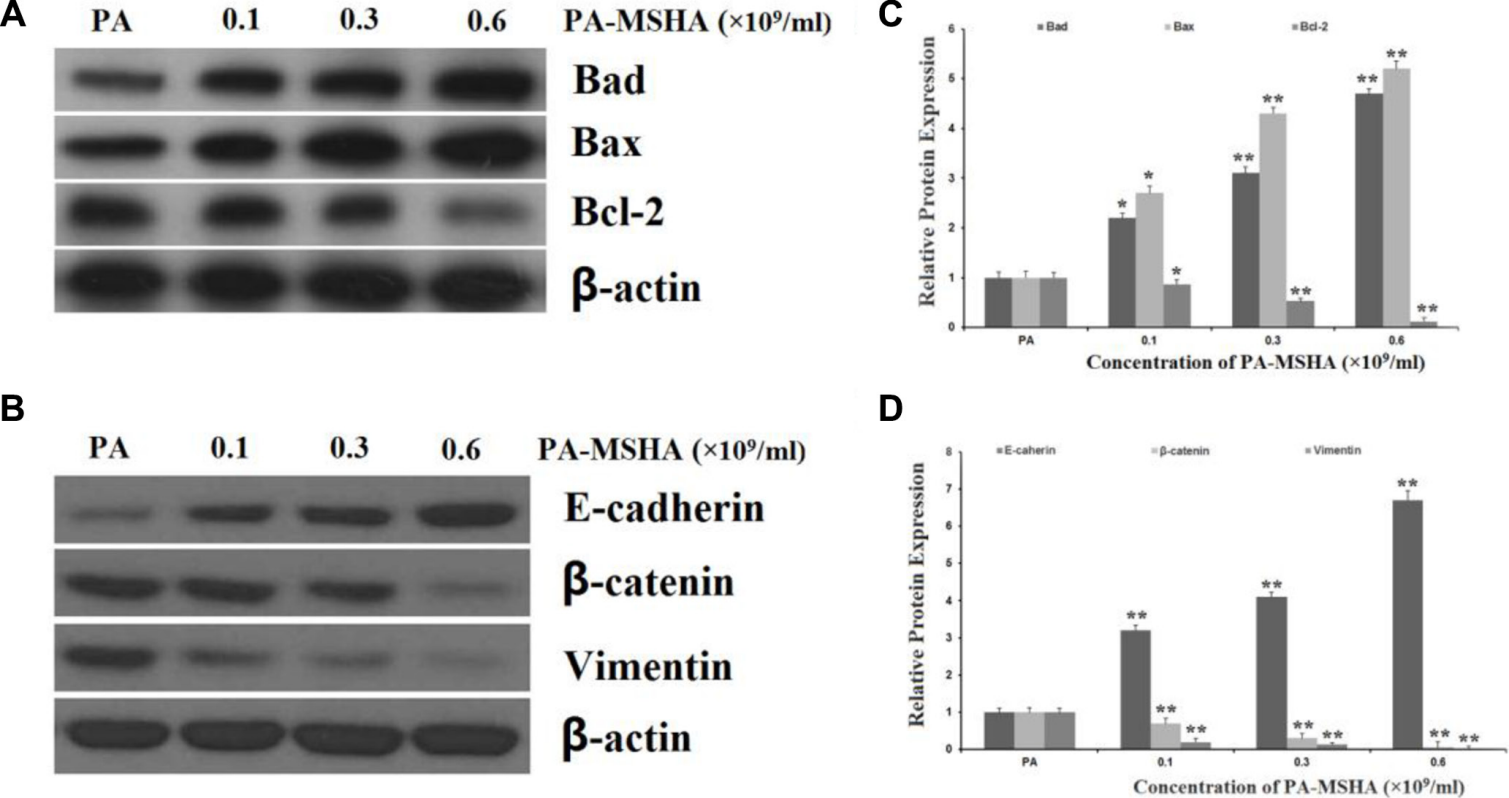
Effects of PA-MSHA on apoptosis- and EMT-related proteins HeLa cells were treated with PA-MSHA or PA. The cells were harvested and the total protein was extracted. (**A**) The effects of PA-MSHA on the protein levels of BAD, BAX and BCL-2 were assessed by Western blot. (**B**) The fold-changes in the relative protein levels of BAD, BAX and BCL-2 were calculated with reference to the control levels. Data are presented as the mean ± SD (*N* = 3, **p < 0.01). (**C**) The effects of PA-MSHA on the protein levels of E-cadherin, Vimentin and β-catenin were assessed by Western blot. (**D**) The fold-changes in the relative protein levels of E-cadherin, Vimentin and β-catenin were calculated with reference to the controls. Data are presented as the mean ± SD (*N* = 3, **p < 0.01).

We next detected the expression of the pro-apoptotic proteins BAD and BAX and the anti-apoptotic protein BCL-2 by Western blotting. As shown in Figure 4A and 4B, at PA-MSHA concentrations of 0.15, 0.3 and 0.6 × 10^9^/mL, the protein expression of BAD and BAX increased and the expression of BCL-2 decreased relative to their expression in PA-treated HeLa cells.

As epithelial-mesenchymal transition (EMT) has been accepted as a potential mechanism underlying cancer cell migration and invasion, we explored the effects of PA-MSHA on EMT-related protein expression in HeLa cells. The addition of PA-MSHA to HeLa cells reduced the expression of Vimentin and β-catenin (mesenchymal markers) and increased the expression of E-cadherin (an epithelial marker) (Figure 4C and 4D).

### PA-MSHA inhibits the activation of the AKT signaling pathway by increasing PTEN expression

**Figure 5:**
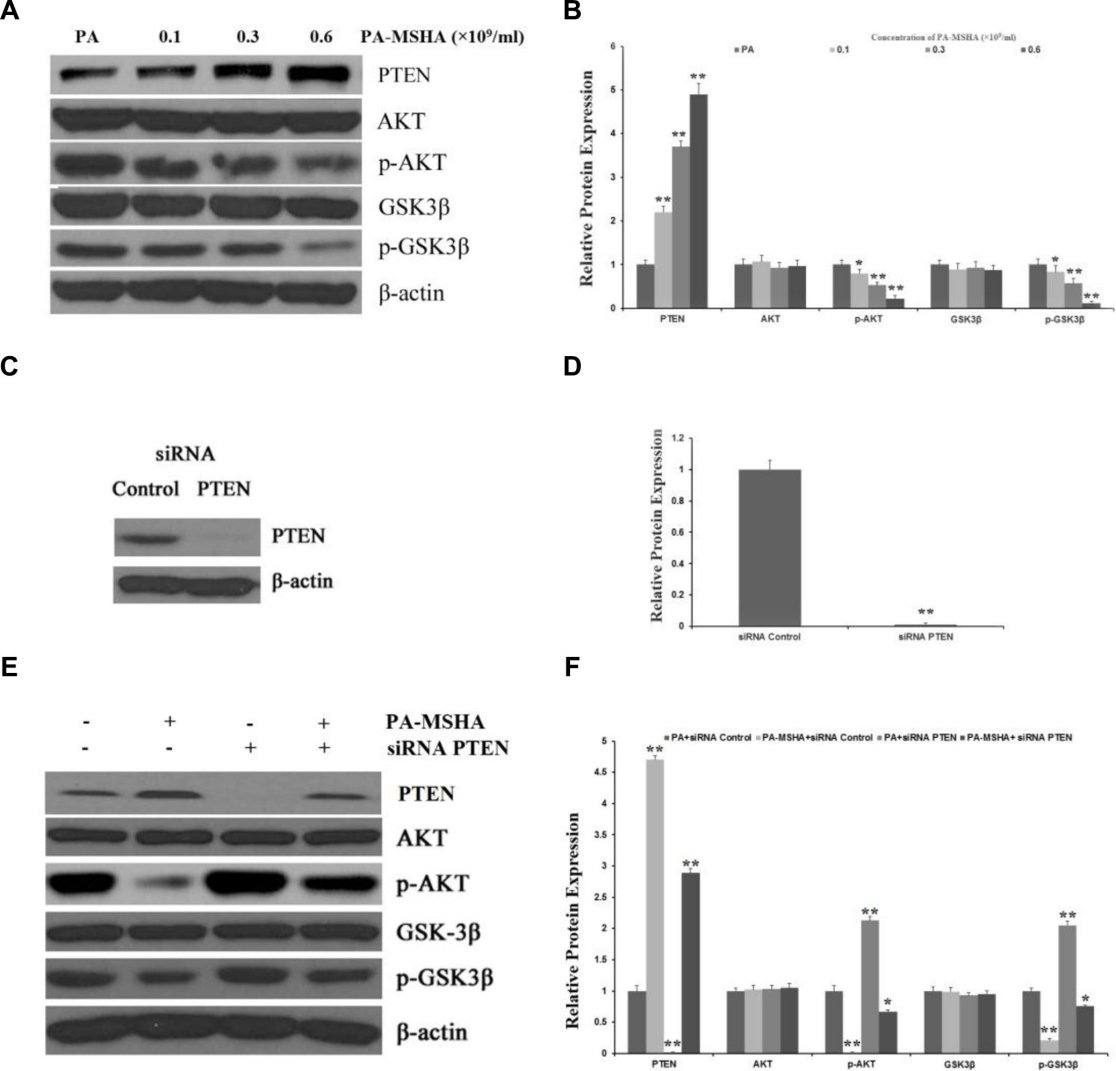
PA-MSHA inhibits the activation of the AKT signaling pathway by increasing PTEN expression HeLa cells were treated with PA-MSHA or PA. The cells were harvested and the total protein was extracted. (**A**) The effects of PA-MSHA on the protein levels of AKT, p-AKT, GSK3β and p-GSK3β were assessed by Western blot. (**B**) The fold-changes in the relative protein levels of AKT, p-AKT, GSK3β and p-GSK3β were calculated with reference to the controls. Data are presented as the mean ± SD (*N* = 3, **p < 0.01). (**C**) Cells were transfected with PTEN siRNA or empty vectors, and Western blot analysis was performed to detect PTEN protein levels. (**D**) The fold-changes in the relative protein levels of PTEN were calculated with reference to the siRNA controls. Data are presented as the mean ± SD (*N* = 3, **p < 0.01). (**E**) The inhibitory effect of PA-MSHA on the *PTEN* siRNA-induced activation of the AKT pathway. (**F**) The fold-changes in the relative protein levels of PTEN, AKT, p-AKT, GSK3β and p-GSK3β were calculated with reference to the controls. Data are presented as the mean ± SD (*N* = 3, **p < 0.01).

Next, we explored the molecular pathways responsible for the effects of PA-MSHA on HeLa cell proliferation and invasion *in vitro*. The phosphatidylinositol-3-kinase (PI3K)/PTEN/AKT pathway is crucial for many aspects of cell growth and survival. Thus, we investigated whether PA-MSHA might inhibit the activation of the AKT signaling pathway by increasing the expression of PTEN. After treatment with PA-MSHA, the expression of PTEN in HeLa cells increased, while the phosphorylation of AKT and its downstream target glycogen synthase kinase 3 beta (p-GSK3β) (Figure 5A and 5B) decreased. These Western blotting results demonstrate that PA-MSHA negatively regulates the AKT pathway by increasing the expression of PTEN.

To further clarify these results, we used siRNA to inhibit the expression of *PTEN* in HeLa cells treated with PA or PA-MSHA (Figure 5C and 5D). While inhibition of *PTEN* significantly increased the expression of p-AKT and p-GSK3β in PA-treated cells, PA-MSHA inhibited the activation of the AKT pathway induced by *PTEN* siRNA (Figure 5E and 5F). These results suggest that the tumor suppression of HeLa cells by PA-MSHA occurs partly through PTEN/AKT signaling.

### PA-MSHA inhibits proliferation and invasion through the PTEN/AKT pathway

**Figure 6:**
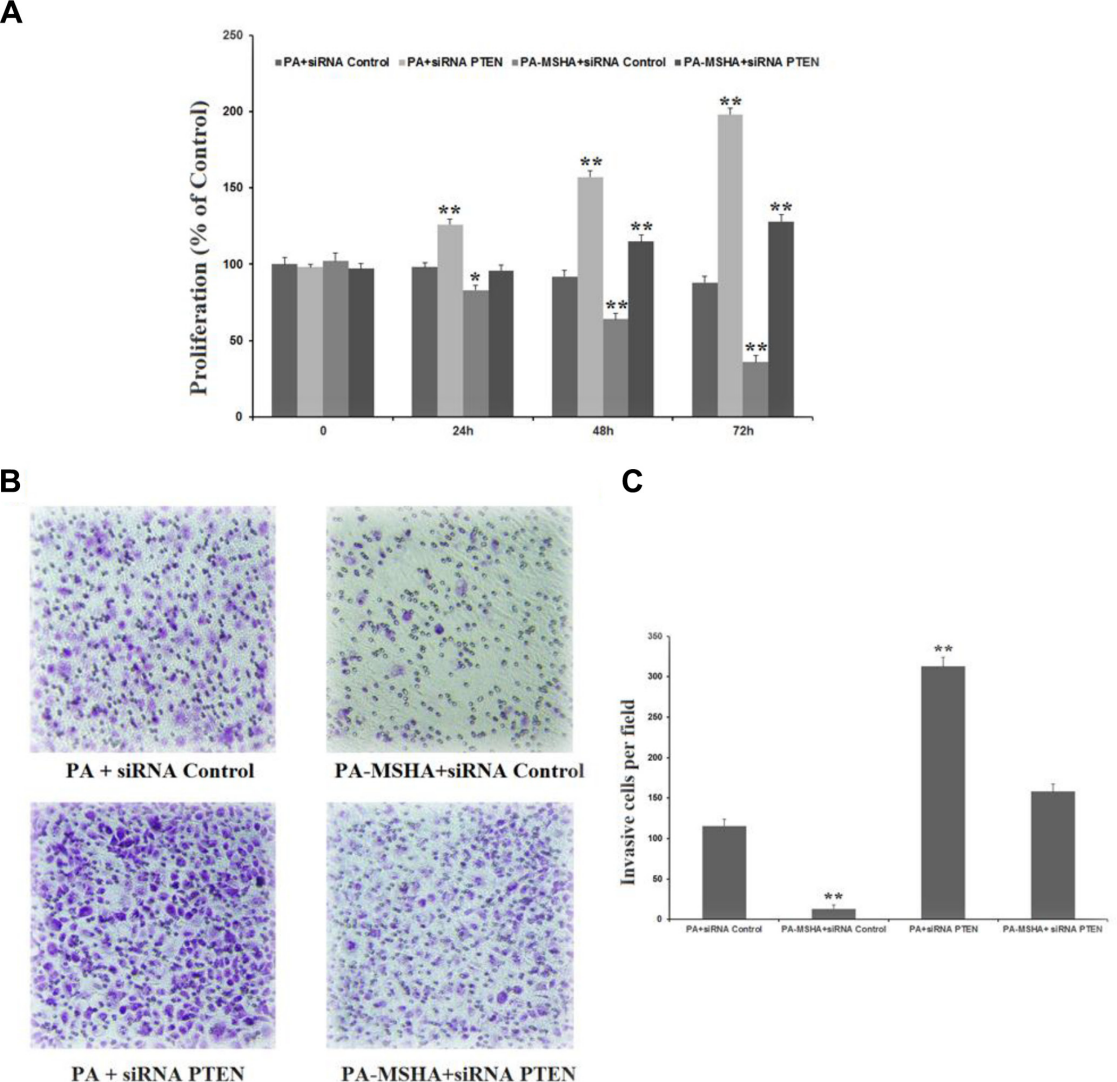
PA-MSHA inhibits proliferation and invasion through the PTEN/AKT pathway (**A**) The inhibitory effect of PA-MSHA on *PTEN* siRNA-induced HeLa cell proliferation. (**B**) The numbers of invasive cells per field were measured in *PTEN* siRNA-treated HeLa cells treated with PA or PA-MSHA, which are depicted in representative pictures. (**C**) The numbers of invasive cells per field were measured in *PTEN* siRNA-treated HeLa cells treated with PA or PA-MSHA. Data are presented as the mean ± SD (*N* = 3, **p < 0.01).

To confirm that PA-MSHA inhibits proliferation and invasion through the PTEN/AKT pathway, we detected the proliferation and invasion of HeLa cells after silencing *PTEN* and treating cells with PA or PA-MSHA. While *PTEN* siRNA increased the proliferation (Figure 6A) and invasion (Figure 6B and 6C) of PA-treated HeLa cells, PA-MSHA inhibited the proliferation and invasion induced by *PTEN* siRNA. These results confirmed that PA-MSHA inhibits proliferation and invasion through the PTEN/AKT pathway.

## DISCUSSION

The PA-MSHA strain is a peritrichous *P. aeruginosa* strain with MSHA fimbriae that was established by Professor Xi-ya Mu. PA-MSHA has been used as an adjuvant therapy in the treatment of patients with malignant tumors to enhance immunity, reduce infection rates and improve overall health [12]. However, studies on the direct anti-cancer cytotoxic effects of PA-MSHA are limited: only five studies have demonstrated these effects of PA-MSHA in hepatocarcinoma, gastric cancer, nasopharyngeal cancer, breast cancer and bladder cancer cell lines [11, 13–15]. Therefore, we sought to explore the cytotoxicity of PA-MSHA in cervical cancer cells. We found that after treatment with PA-MSHA, HeLa cells were arrested in G2/M phase, and apoptosis was induced in a dose-dependent manner. In addition, PA-MSHA impaired the migration and invasion abilities of HeLa cells.

Proteins from the BCL-2 family can either positively or negatively regulate apoptosis. The anti-apoptotic members of this family include BCL-2, BCL-XL and BCL-w, while the pro-apoptotic family members include BAX, BAD and BOK [18, 19]. We examined the effects of PA-MSHA on the expression of BAD, BAX and BCL-2 by Western blot analysis. PA-MSHA induced BAD and BAX and inhibited BCL-2, suggesting that PA-MSHA promotes apoptosis in cervical cancer cells by increasing the expression of pro-apoptotic genes and reducing the expression of anti-apoptotic genes.

EMT is known to be a central mechanism responsible for the invasiveness and metastasis of various cancers. Thus, we determined the expression of mesenchymal markers (Vimentin and β-catenin) and an epithelial marker (E-cadherin) in HeLa cells. PA-MSHA reduced the expression of Vimentin and β-catenin and increased the expression of E-cadherin, suggesting that PA-MSHA can suppress EMT.

Next, we tried to elucidate the molecular pathways through which PA-MSHA inhibits HeLa cell proliferation and invasion *in vitro*. PTEN/AKT signaling is frequently activated in various cancers and is critical for promoting proliferation and invasion [16]. PTEN is a tumor suppressor that downregulates AKT signaling by reducing the output of PI3K at the cell membrane. GSK3β is constitutively active, but can be inactivated through the phosphorylation of a single residue at serine 9 by AKT. GSK-3β actively promoted cell growth, proliferation, survival, gene expression, differentiation etc [17]. Now, we have demonstrated that PA-MSHA can reduce the phosphorylation of AKT and GSK-3β by increasing the expression of PTEN. We also found that treatment of HeLa cells with PTEN siRNA significantly increased the expression of p-AKT and p-GSK3β, but further treatment with PA-MSHA suppressed the activation of the AKT pathway induced by PTEN siRNA. Furthermore, we confirmed that PTEN siRNA increased the proliferation and invasion of HeLa cells, while PA-MSHA inhibited the proliferation and invasion induced by PTEN siRNA. Based on all these results, we propose a novel mechanism by which PA-MSHA suppresses the proliferation and invasion of cervical cancer cells: by activating PTEN and thus inhibiting the AKT/GSK3β pathway.

In summary, our data indicate that PA-MSHA has anti-proliferative/chemotherapeutic effects on cervical cancer cells and inhibits their invasion by influencing the PTEN/AKT pathway. The present study is the first to assess the cytotoxic effects of PA-MSHA in cervical cancer cells. These experiments with PA-MSHA indicate that bacteria can induce cancer regression not only by activating the immune system, but also by inducing cytotoxicity. We propose that the use of PA-MSHA, either alone or in combination with standard therapy, could be a novel strategy for the management of cervical cancer. However, further studies are needed to validate the present findings in appropriate animal models.

## MATERIALS AND METHODS

### Cell lines and materials

The human cervical carcinoma (HeLa) and human cervical epithelial (H8) cell lines were obtained from the Institute of Basic Medical Sciences of the Chinese Academy of Medical Sciences (Perking, China). PA-MSHA was purchased from Beijing Wanteer Bio-Pharmaceutical Co., LTD (Perking, China). *P. aeruginosa* (PA) was provided by the China General Microbiological Culture Collection Center (Perking, China). All antibodies were purchased from Santa Cruz Biotechnology (CA, USA). RPMI-1640 and trypsin were obtained from Gibco(Grand Island, NY, USA). The SuperScript^TM^ First-Strand cDNA Synthesis System was purchased from Promega (Madison, WI, USA). The Annexin V-fluorescein isothiocyanate (FITC) Apoptosis Detection Kit was obtained from the Beyotime Institute of Biotechnology (Jiangsu, China).

### MTT assay

The MTT assay was used to measure cell proliferation. Briefly, cells (4 × 10^3^) were seeded in 96-well culture plates in a concentration- or time-dependent manner. The following day, the media were removed, and fresh media containing 1% fetal bovine serum (FBS) and several concentrations of PA-MSHA or PA (4.8, 2.4, 1.2, 0.6, 0.3 and 0.15 × l0^9^ bacteria/mL) were added. The cells were then incubated at 37°C for 0, 24, 48, or 72 h. For time-dependent manner, the concentration of PA-MSHA was 0.6 × l0^9^ bacteria/mL. PA (0.3 × 10^9^bacteria/mL) was added as a negative control, and the cells were then incubated at 37°C for up to 5 days. Subsequently, 0.025 mL of MTT solution (5 mg/mL) was added to each well, and the cells were incubated for another 4 h. After centrifugation, the supernatant was removed from each well. The colored formazan crystals produced from MTT in each well were dissolved in 0.15 mL of DMSO, and the optical density (OD) values were measured at 490 nm.

### Flow cytometric analysis of the cell cycle

The cells were treated with the indicated concentrations of PA (0.3 × 10^9^/mL) or PA-MSHA (0.15, 0.3 or 0.6 × 10^9^/mL) for 48 h. Next, the cells were harvested and washed twice with PBS. The cells were fixed with 70% ethanol overnight and were harvested and centrifuged at 1500 g for 5 min. Then, the cell density was adjusted to 1 × 10^6^ cells/mL with PBS. Finally, the cells were stained with a propidium iodide (PI) solution (50 μg/mL). The number of cells in each phase of the cell cycle was analyzed by a FACStarflow cytometer (BD Bioscience, San Jose, CA, USA).

### Flow cytometric analysis of cell apoptosis

The cells were treated with PA (0.3 × 10^9^/mL) or PA-MSHA (0.15, 0.3 or 0.6 × 10^9^/mL) for 48 h, and viable cells were harvested and washed twice with PBS. Viable cells were double-stained with FITC-conjugated Annexin V and PI. Apoptosis was analyzed by quadrant statistics. The Annexin V-FITC was highlighted in green, and PI was highlighted in red.

### Wound-healing assay

Cells were plated and grown overnight to confluence on a six-well plate. Monolayers of cells were wounded by manual scraping with a 10-μL pipette tip. Cells were rinsed with PBS and then treated with PA (0.3 × 10^9^/mL) or PA-MSHA (0.15, 0.3 or 0.6 × 10^9^/mL) in serum-free medium. Images were taken at 0 and 24 h after wounding under an inverted microscope.

### Cell invasion assay

The cells were cultured in serum-free medium for 24 h, and then same numbers of cells were seeded onto the basement membrane matrix (EC matrix, Chemicon, Temecula, CA, USA) in the insert of a 24-well culture plate. FBS was added to the lower chamber as a chemoattractant. The cells were treated with PA (0.3 × 10^9^/mL) or PA-MSHA (0.15, 0.3 or 0.6 × 10^9^/mL). After 12 h, the non-invading cells and the EC matrix were gently removed with a cotton swab. The invasive cells located on the lower side of the chamber were stained with Crystal Violet, air-dried and photographed. Cell migration was quantified by direct microscopic visualization and counting. The values for invasion were obtained through the counting of cells in three fields per membrane. The results are presented as the average of six independent experiments performed over multiple days.

### SiRNA and transfection

To knock down the expression of Phosphatase and tensin homolog (*PTEN*) in HeLa cells, we transfected small interfering RNA (siRNA) of *PTEN* or the negative control (NC) (Genepharma, Shanghai, China) into HeLa cells using lipofectamine 2000 (Invitrogen, Carlsbad, CA, USA) according to the manufacturer's protocol. Transiently transfected cells were harvested after 48 h for protein analysis.

### Western blot analysis

After the cells were treated with PA (0.3 × 10^9^/mL) or PA-MSHA (0.15, 0.3 or 0.6 × 10^9^/mL) for 24 h, the proteins were isolated with cell lysis buffer. The protein concentrations were determined with a BCA protein assay kit (Pierce, USA). Then, 30 μg of the total lysate was separated by SDS-PAGE under standard conditions and transferred onto a PVDF membrane for Western blot analysis. The antibodies used for detection included rabbit anti-PTEN, anti-AKT, anti-p-AKT, anti-GSK3β, anti-p-GSK3β, anti-BCL-2, anti-BAX, anti-BAD, anti-E-cadherin, anti-Vimentin and anti-β-catenin, and mouse anti-β-actin. The membranes were washed and incubated with the appropriate secondary antibody (anti-mouse or anti-rabbit IgG). The immunoreactive proteins were detected with an ECL detection reagent and were captured on X-ray film. β-actin was used as a control for protein loading.

### Statistical analyses

The data from these experiments were assessed with SPSS 13.0. Differences between groups were determined with Student's *t*-test or one-way analysis of variance (ANOVA). Values of *p* < 0.05 were considered to be statistically significant.
